# Continued Use of Contact-Tracing Apps in the United States and the United Kingdom: Insights From a Comparative Study Through the Lens of the Health Belief Model

**DOI:** 10.2196/40302

**Published:** 2022-12-08

**Authors:** Zhan Zhang, Isaac Vaghefi

**Affiliations:** 1 School of Computer Science and Information Systems Pace University New York, NY United States; 2 Zicklin School of Business Baruch College City University of New York New York, NY United States

**Keywords:** contact tracing, app adoption, app continued use, public attitudes, health belief model, COVID-19

## Abstract

**Background:**

To contain the spread of SARS-CoV-2, contact-tracing (CT) mobile apps were developed and deployed to identify and notify individuals who have exposure to the virus. However, the effectiveness of these apps depends not only on their adoption by the general population but also on their continued use in the long term. Limited research has investigated the facilitators of and barriers to the continued use of CT apps.

**Objective:**

In this study, we aimed to examine factors influencing the continued use intentions of CT apps based on the health belief model. In addition, we investigated the differences between users and nonusers and between the US and UK populations.

**Methods:**

We administered a survey in the United States and the United Kingdom. Respondents included individuals who had previously used CT technologies and those without experience. We used the structural equation modeling technique to validate the proposed research model and hypotheses.

**Results:**

Analysis of data collected from 362 individuals showed that perceived benefits, self-efficacy, perceived severity, perceived susceptibility, and cues to action positively predicted the continued use intentions of CT apps, while perceived barriers could reduce them. We observed few differences between the US and UK groups; the only exception was the effect of COVID-19 threat susceptibility, which was significant for the UK group but not for the US group. Finally, we found that the only significant difference between users and nonusers was related to perceived barriers, which may not influence nonusers’ continued use intentions but significantly reduce experienced users’ intentions.

**Conclusions:**

Our findings have implications for technological design and policy. These insights can potentially help governments, technology companies, and media outlets to create strategies and policies to promote app adoption for new users and sustain continued use for existing users in the long run.

## Introduction

### Overview

The COVID-19 outbreak, a consequence of SARS-CoV-2, has had a major, long-lasting effect on our society. The COVID-19 pandemic has been considered the most significant public health threat the world has experienced in the last 100 years [1]. In the United States alone, there have been over 80 million reported cases of the disease, with over a million fatal cases [2]. The COVID-19 pandemic has lasted for over 2 years and several new, high-transmissibility variants (eg, delta and omicron) have emerged during this time. Various efforts (eg, the distribution of multiple vaccines) and containment measures have been taken worldwide, including social distancing, travel restrictions, testing, and contact tracing (CT). Among these measures, digital CT apps have been considered a particularly important strategy for managing the pandemic and reducing COVID-19 cases and deaths [3,4]. These apps can rapidly identify and notify exposures to the disease [5,6].

Although traditionally performed manually [7], a set of CT apps have been developed and deployed by different countries (eg, China, the United States, the United Kingdom, Japan, Israel, and Singapore), health providers (eg, Mayo Clinic), and technology companies (eg, Apple and Google). Regardless of the differences in app design and architecture, prior research has indicated that the effectiveness of these apps is proportional to the number of people who use them [8]; that is, to suppress the epidemic, 80% of all smartphone users or 56% of the population overall need to use the CT app [3,9]. A survey conducted early in the pandemic reported that ≥60% of the population indicated a willingness to install a hypothetical CT app [10]. Yet, the actual adoption of these apps has been considerably lower than anticipated [11], with installation rates of ≤10% of the population in some countries where apps have been deployed [12]. This fact highlights the intention-behavior gap [13]—even though many people may have displayed the intention to use a CT app—they do not take action by installing or using it. Such low adoption rates are problematic and prevent the actual value of CT apps from being realized.

According to the World Health Organization, several other diseases could likely cause pandemics in the future, which signifies the importance of preparing for such occasions, even after the current pandemic is over [14]. Therefore, as an effective approach to containing pandemics, there is a need to understand why CT apps have not been as popular as anticipated and what factors facilitate or hinder their continued use as pandemics go on. Investigating this problem is critical not only for addressing the COVID-19 pandemic but also for understanding how to design CT technologies and apps for future health crises.

Ample research has been conducted in the early phase of the COVID-19 pandemic to investigate factors that could influence the adoption and uptake of CT apps [15]. For instance, prior work pointed to the influence of demographics (eg, age and sex) [16-18], individual beliefs and attitudes (eg, trust, privacy concerns, or access to technologies) [10,19,20], situational factors (eg, COVID-19 cases and deaths or lockdown measures) [21], and contextual factors (eg, cultural, regional, and national differences) [15]. Despite the important contributions made by these studies, as Jamieson et al [22] stated, “the collective utility of contact tracing technology to suppress the spread of viruses depends not only on the adoption of contact tracing apps but also on their continued use.” In addition, compared with the beginning of the pandemic, our society has gained more knowledge about COVID-19 and a significant portion of the population has been vaccinated, all of which could affect people’s willingness to continue the use of CT apps. More importantly, recent evidence in health informatics shows that sustained and continued use of CT apps may have different motivators than the initial adoption of these technologies [23,24]. To our knowledge, *there is limited research that pays scholarly attention to the continued use of CT apps* [25].

Building upon prior work in this domain, we aim to address this research gap by proposing and validating a research model of the predictors of continued use of CT apps, defined as extending the use of CT apps beyond the initial stages of adoption and the first few uses. Our specific research questions include the following: (1) What are the predictors of an individual’s continued intention to use CT apps? (2) How do user behaviors and predictors differ among users who had experience versus those who did not and between individuals in the United States versus the United Kingdom? To answer these research questions, we surveyed respondents in the United States and the United Kingdom from among those who had previously used CT technologies and those without any experience. Our findings highlighted several factors (eg, perceived benefits, self-efficacy, perceived severity, perceived susceptibility, and cues to action) that have positive impacts on the continued use of CT apps. In contrast, perceived barriers could reduce people’s continued use intentions. We also observed some differences between the United States and the United Kingdom and between users and nonusers. Our study could provide important insights for governments, technology companies, and media outlets to determine how to promote CT apps better and sustain continued use in the long run.

### Background

Since the outbreak of the COVID-19 pandemic, seminal research has studied user acceptance and use intention of CT apps based on several theoretical models, such as the health belief model (HBM) [26], the protection motivation theory [27], health behavior change [28], cognitive appraisal theory [29], procedural fairness theory and cultural dimension theory [19], and the unified theory of acceptance and use of technology [30-32]. These studies primarily focused on evaluating the public’s attitude; for example, individuals’ willingness or intention to install and adopt a hypothetical CT app.

This body of prior work highlighted a set of factors that influence the adoption and use intention of CT apps, ranging from individual characteristics (eg, age, sex, and experience with technology) to situational (eg, lockdown measures) and contextual factors (eg, cultural and national differences). For example, younger ages [10,16,17], higher education level [18,26,33], and experience using smartphone apps [10,34] are associated with positive intentions to download a CT app. In addition, the perceptions, trust, and acceptance of CT technologies seem to vary in different countries and cultural backgrounds. For example, countries with collectivist values, such as those in Asian regions, usually see broad acceptance from their citizens [10,16]; in contrast, the US and European respondents generally report lower acceptance [10,17,18,35]. Situational factors, such as the total number of COVID-19 cases in a region and lockdown measures, could also affect people’s willingness to download the CT app [21,25]. For example, those living in places with lockdown measures or restrictions on mobility are less likely to download and use the app [25].

In summary, prior work has primarily investigated the acceptance and intention to adopt and use CT technology. This is because most of the reviewed studies were conducted before or shortly after the introduction of CT apps; therefore, they were only able to measure people’s willingness or intention to use a newly developed CT app. In addition, although some studies examined the actual use of CT apps with users [17,20,36,37], they only explored adoption at an early stage. *Thus,* *continued use remains to be investigated* [22,25]. To that end, in this study, we examined the continued use of CT apps beyond the initial stages of adoption and the first few interactions. We included both prior app users and nonusers in our study to investigate the factors that impact a person’s continuous intention to use CT apps and how the influence of key predictors may differ between the user and nonuser groups.

### Theoretical Framework and Hypotheses Development

In this work, we examine which factors influence an individual’s *continued use* of a CT app by adopting the HBM [38,39]. We chose this theoretical model because the HBM is a widely recognized model in the context of health behavior change [38] and has been used by many prior studies to explain why people follow healthy choices of action in the presence of a threat. This is comparable with our study’s context, where individuals may continue to use a CT app as a behavior to help counteract the threat of contracting and spreading COVID-19.

The HBM consists of several constructs including perceived susceptibility, perceived benefits, perceived barriers, perceived severity, and cues to action. The model assumes that people who anticipate a health threat are more willing to perform a protective health behavior because they believe that such an action will reduce a severe illness [40,41]. The 2 constructs of the HBM—perceived susceptibility and perceived severity—are highly related to this cognitive presumption. *Perceived susceptibility* represents an individual’s perceived risk or likelihood of catching a disease due to a particular behavior. *Perceived severity* refers to individuals’ beliefs about the impact of the harm of pursuing a particular behavior. Several studies have revealed that perceived susceptibility and perceived severity can cause people to take protective actions, such as using and adopting mobile health technologies [42,43]. In relation to the current COVID-19 pandemic, if people perceive themselves as liable to COVID-19 infection and related health complications, they are more inclined to continue using the app to reduce the infection risk of COVID-19. In addition, someone who perceives high health threats and severity tends to continue using the CT app. Therefore, we hypothesized the following:

Hypothesis 1: The perceived susceptibility to COVID-19 is positively associated with the continued use intention of CT apps.

Hypothesis 2: The perceived threat of COVID-19 is positively associated with the continued use intention of CT apps.

*Perceived benefits* refer to a person’s belief that recommended health behaviors will be beneficial in preventing the disease or reducing its effect. A high perception of benefits increases the likelihood of adopting such behavior. *Perceived barriers*, on the contrary, represent the costs of or obstacles to performing the recommended health behavior, including tangible costs (eg, time, money, and knowledge acquisition) and psychological costs (eg, feeling anxious, pessimistic, and embarrassed) [44]. Low perception of barriers increases a person’s willingness to adopt a particular behavior. The model assumes that the more benefits the individual believes there are from a new behavior, and the lower the obstacles to performing such behavior, the greater the chance of adopting it [45]. In our study context, perceived benefits could include personal benefits (eg, being formed of a potential infection to protect a person’s health) and social benefits (eg, helping the community contain the spread of the coronavirus). Perceived barriers, as pointed out in prior works [46,47], include privacy issues and security concerns raised by the app. These concerns could present barriers to using the app in the long term. Therefore, we hypothesized as follows:

Hypothesis 3: The perceived benefits of CT apps are positively associated with the continued use intention of CT apps.

Hypothesis 4: The perceived barriers of CT apps are negatively associated with the continued use intention of CT apps.

The HBM is often complemented by constructs and factors related to health and protective behavior [48]. Therefore, we added perceived self-efficacy to the model, as we believe that the continued use of CT apps is also influenced by individuals’ beliefs in their competence to use the app. *Perceived self-efficacy* is an individual’s belief that he or she can successfully perform a particular health behavior. A few studies have demonstrated that self-efficacy is a significant factor in predicting health behaviors [49,50]. In addition, research has addressed the role of self-efficacy in predicting users’ intention to continue using information technology systems [51]. In the context of COVID-19, if individuals have found mastery of the app, they may be more inclined to adopt the app [19,26,27]. However, whether having the ability to use CT apps could predict continued intention is understudied. Therefore, we hypothesized the following:

Hypothesis 5: Perceived self-efficacy is positively associated with the continued use intention of CT apps.

*Cues to action* are the circumstances that inspire the readiness to act. This construct can influence individuals’ decisions on whether to engage in protective behaviors. Concerning COVID-19, cues to action include exposure to media content and the infection experience of close friends and family members. It has been 2 years since the outbreak of the pandemic when this study was performed. People, especially existing users of CT apps, have had a variety of ways to get to know about COVID-19 and experience CT technology. Therefore, they may be more inclined to continue using the app. Our hypothesis was as follows:

Hypothesis 6: Cues to action are positively associated with the continued use intention of CT apps.

Textbox 1 provides an overview of the model constructs and their definitions in this study. In addition to these constructs, we included several factors that may influence the behavioral decision to use the app, such as individual characteristics (eg, age, sex, or educational level), contextual differences (United States vs United Kingdom), and COVID-19 experiences (whether they previously contracted COVID-19).

**Overview of contextualized constructs according to the health belief model.**

- Perceived benefits of contact-tracing (CT) app use: perceptions about the positive outcomes of using CT apps.
- Perceived barriers of CT app use: perceptions about the negative outcomes or obstacles of using CT apps.
- COVID-19 threat severity: the extent to which one’s health might be negatively affected by the COVID-19 pandemic.
- COVID-19 threat susceptibility: the extent to which one feels vulnerable to contracting COVID-19.
- Self-efficacy to use CT apps: belief in having the resources, skills, and ability to continue using CT apps.
- Cue to action (using CT apps): cues that trigger the use of CT apps.
- (CT apps) Continued use intention: willingness to continue the use of a mobile health app [52].

## Methods

### Data Collection

Pursuant to our research questions, we conducted a survey study among individuals in the United States and the United Kingdom to evaluate their intentions to continue using CT apps. We conducted the surveys in fall 2021 (October to December). To be open in our data collection and insights, we surveyed individuals who previously used CT technologies, in addition to those who have had no prior experience. This helped us gauge responses from both users and nonusers to examine what would generally determine decisions for long-term engagement with CT apps.

Individuals were recruited with the help of 2 survey companies: Amazon Mechanical Turk in the United States and Prolific in the United Kingdom. We used a web-based survey (Qualtrics) to collect the responses. No identifying information was collected to ensure anonymity. Nonetheless, we collected demographic information as shown in Table 1. To calculate the minimum sample size necessary for variance-based structural equation model analysis, we followed the study by Hair et al [53], which suggests a sufficient sample that is a minimum of 10 times the number of items of the formative indicators in the model. Given that we had 24 items formatively represented in the model, we concluded that a minimum sample size of 240 was required.

Of the 532 individuals who initiated the surveys, 363 (68.2%; Table 1) completed them (171 US and 203 UK respondents). The response rate is acceptable, given the sensitive nature of some questions [54]. Yet, we checked for nonresponse bias by comparing the demographic characteristics of respondents and nonrespondents; the results showed no significant differences, suggesting that nonresponse bias was not an issue in our study.

The measurement items were selected from previously validated measures [26,55,56] and adapted to fit the study context (see Table 2 for measurement items). Although the measurements were validated in prior research, we conducted a pilot study and asked a convenience sample of 12 CT app users to review and reflect on the survey and provide qualitative feedback regarding the questionnaire guide measurement instruments. We also asked them to provide written feedback on the appropriateness, readability, and meaningfulness of the measures and their fit to the context of the study. On the basis of the qualitative feedback provided, we revised the survey guidelines to avoid possible confusion in responding and introducing any bias. Furthermore, we have slightly reworded a few items for clarity. For instance, item 1 in perceived barriers was changed from “The contact tracing app will violate my rights” to “The contact tracing app will violate my privacy.” Overall, the pilot study helped us improve the quality and accuracy of the data collection instrument.

**Table 1:** Demographic characteristics.

		Values (N=363), n (%)
**Age (years)**		
	18-25	50 (14)
	25-35	119 (33)
	35-45	99 (27)
	45-55	66 (18)
	55-78	29 (8)
**Sex**		
	Male	160 (44)
	Female	200 (55)
	Nonbinary	3 (1)
**Education**		
	Less than high school	6 (2)
	High school graduate	45 (12)
	Some college	85 (23)
	2-year degree	44 (12)
	4-year degree	138 (38)
	Professional degree	40 (11)
	Doctorate	5 (1)
**Contact-tracing app experience**		
	Currently using	125 (56)
	Used in the past	114 (16)
	Never used	124 (22)

**Table 2:** Measurement items and loadings.

Variable and items				Loadings
**Perceived benefits of contact-tracing apps [26]**				
	Thanks to the contact-tracing app, I will be more on my guard when I have face-to-face contact.		0.785	
	Thanks to the contact-tracing app, I will take more precautions not to spread the Coronavirus myself (eg, wash my hands, maintain distance from others, and limit my outside movements).		0.719	
	By using the contact-tracing app, I will help public authorities combat the Coronavirus.		0.732	
	The contact tracing app will allow me to protect myself from the Coronavirus.		0.752	
**Perceived barriers of contact-tracing app use [26]**				
	The contact-tracing app will violate my privacy.		0.855	
	The contact-tracing app will create tensions between individuals who are infected by the Coronavirus and those who are not.		0.91	
**COVID-19 threat severity [26]**				
	If I get infected by the Coronavirus, it will have important health consequences for me.		0.843	
	If I get infected by the Coronavirus, my health will be severely affected.		0.895	
	If I get infected by the Coronavirus, my health will be significantly reduced.		0.914	
**COVID-19 threat susceptibility [26]**				
	I am at risk of being infected by the Coronavirus.		0.826	
	It is likely that I would suffer from the Coronavirus.		0.649	
	It is possible that I could be infected by the Coronavirus.		0.738	
**Self-efficacy to use contact-tracing apps [57]**				
	I have the knowledge needed to use the contact-tracing app.		0.914	
	I have the necessary resources to use the contact-tracing app.		0.915	
	I can get help from others if I experience difficulties using the contact-tracing app.		0.724	
**Cue to action (using contact-tracing apps) [26]**				
	**To what extent do the following cues prompt the use of a contact-tracing app?**			
		Hearing someone near you contracted COVID-19	0.712	
		Website of a newspaper, TV or radio station, or magazine	0.938	
		App of a newspaper, TV or radio station, or magazine	0.984	
		News shared on social media (Facebook, YouTube, Twitter, Instagram, WhatsApp, etc)	0.901	
		Alerts through email and newsletters	0.908	
**(Contact-tracing apps) Continued use intention [52]**				
	I would be willing to continue using contact-tracing app.		0.912	
	I plan to continue using contact-tracing app.		0.920	
	I want to continue using contact-tracing app in the future.		0.901	
**Control factors**				
	Demographic factors: age, sex, education		—^a^	
	COVID-19 experience: Have you or a person close to you (ie, a close friend or family) been affected by COVID-19?		—	
	Contact-tracing app experience		—	

^a^Not available.

### Data Analysis

Before testing the model, we assessed reliability and validity (ie, interconstruct correlations, Cronbach *α* values, composite reliability, and average variance extracted), as well as descriptive statistics (Table 3). These variables showed good reliability. The *α* and composite reliability scores were all above the 0.7 acceptable thresholds [58]. The average variance extracted scores were also above 0.5, indicating good convergent validity (ibid). Furthermore, the square roots of average variance extracted scores (on the matrix’s diagonal) were higher than the correlations with the other constructs. In addition, the item loadings (see Table 2, last column) were all above 0.7 and loaded primarily onto their factor, which confirms a good discriminant validity [58]. Altogether, the results supported the reliability and validity of the constructs [59].

Subsequently, we used the structural equation modeling technique to validate the proposed research model and hypotheses. Finally, we ran additional analyses to measure the differences between users and nonusers and between-country differences. More details about these analyses are provided in the *Results* section.

**Table 3:** Descriptive statistics, correlations, reliability, and validity.

Constructs	Value, mean (SD)	Cronbach *α*	CR^a^	AVE^b^	1	2	3	4	5	6	7
1. Perceived benefits of CT^c^ app use	4.40 (1.60)	.91	0.94	0.79	0.89	—^d^	—	—	—	—	—
2. Perceived barriers of CT app use	4.17 (1.58)	.71	0.87	0.77	−0.44	0.88	—	—	—	—	—
3. COVID-19 threat severity	4.22 (1.20)	.85	0.92	0.90	0.22	−0.10	0.90	—	—	—	—
4. COVID-19 threat susceptibility	4.72 (1.44)	.85	0.91	0.77	0.22	−0.21	0.49	0.88	—	—	—
5. Self-efficacy to use CT apps	4.72 (1.12)	.79	0.88	0.71	0.24	−0.08	−0.02	0.03	0.84	—	—
6. Cue to action (using CT apps)	2.61 (1.11)	.92	0.94	0.76	0.70	−0.44	0.22	0.31	0.20	0.87	—
7. (CT apps) Continued use intention	4.28 (2.03)	.98	0.99	0.96	0.72	−0.55	0.13	0.29	0.25	0.74	0.98

^a^CR: composite reliability.

^b^AVE: average variance extracted.

^c^CT: contact tracing.

^d^Not applicable.

### Ethics Approval

This study was approved by the institutional review board of Pace University (IRB number 172765) before conducting any data collection.

## Results

### Structural Model Testing

**Figure 1:**
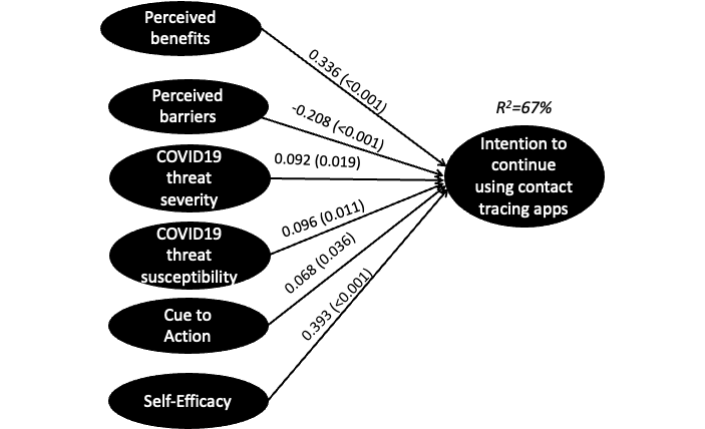
Results of the structural equation modeling.

We used a variance-based structural equation model analysis with SmartPLS (version 3; SmartPLS) to test the proposed research model. The results (Figure 1) supported the proposed hypotheses. We found that the perceived benefits of CT apps contribute to higher continued use intention (*β*=.336; *P*<.001), while the perceived barriers can reduce individuals’ willingness to do so (*β*=−0.208; *P*<.001). We also found that the perceptions of severity (*β*=.092; *P*=.02) and susceptibility (*β*=.096; *P*=.01) to COVID-19 as a significant health threat positively predict one’s continued use intention. Next, we found that having self-efficacy to use CT apps, that is, being confident about having the knowledge and skills required to work with the app, can positively explain continued use intentions (*β*=.393; *P*<.001). Finally, cues from news, media, and peers can increase continued use intentions (*β*=.068; *P*=.03). Together, these factors explained 67% of the variance of the intention to continue using a CT app.

Although the direct hypothesized relationships were significant, the control variables (eg, age, sex, or education) posed no significant effect on the model and hypothesized relationships.

### Post Hoc Analysis 1: Testing Country-Based Differences

We performed additional analyses to check for heterogeneity in individuals’ CT app use. We tested the possible differences between app users’ intentions and their predictors (1) among individuals in the United States (n=160) versus the United Kingdom (n=203) and (2) among those who had previously used CT apps (n=239) versus those who did not (n=124). Accordingly, we followed the multigroup analysis (MGA) procedure [60] in PLS, which allows for direct nonparametric testing of the path estimates in the structural model for each bootstrap sample.

One potential concern when running an MGA is the measurement invariance [61]; hence, it is important to assess whether the construct measures are invariant between the samples [60]. To establish measurement invariance, we checked the difference in item loadings across the 2 samples and whether the difference is statistically significant. Following the procedure by Henseler et al [62], all *P* values were above .05, except for 1 item (Benefits_4), which we dropped from further analysis (leaving 3 other reflective items for that construct). Next, we tested the differences in the complete structural model (Table 4). The results showed that the influence of predictors is largely similar in both the US and UK groups. Yet, we found a significant difference regarding the effect of COVID-19 threat susceptibility (*P*=.01), as it significantly contributed to continued use intention for the UK group but not for the US group. In addition, when considering each group individually, we found nonsignificant effects for COVID-19 threat severity and susceptibility among the US group and self-efficacy to use CT apps for the UK group.

**Table 4:** Results of post hoc analysis 1.

Predictor: (CT^a^ apps) continued use intention	Path coefficients (United States)	*P* value (United States)	Path coefficients (United Kingdom)	*P* value (United Kingdom)	Path coefficients (difference)	*P* value (difference)
Perceived benefits of CT app use	0.32	<.001	0.36	<.001	−0.04	.74^b^
Perceived barriers of CT app use	−0.21	<.001	−0.19	<.001	−0.02	.77^b^
COVID-19 threat severity	0.04	.51^b^	0.12	.01	−0.08	.29^b^
COVID-19 threat susceptibility	−0.02	.71^b^	0.19	<.001	−0.21	.01
Self-efficacy to use CT apps	0.14	.01	0.01	.85^b^	0.13	.06^b^
Cue to action (using CT apps)	0.41	<.001	0.38	<.001	0.04	.76^b^

^a^CT: contact tracing.

^b^Not significant.

### Post Hoc Analysis 2: Testing Experience-Based Differences

In the second post hoc analysis, we checked for potential differences in findings based on individuals’ prior experience with CT apps. Following the same procedure, we assessed the measurement item invariance using the MGA procedure in PLS. We found that 2 items (Benefit_4 and CuestoAction_2) were not invariant across samples and hence were dropped before the MGA. Next, we tested for significant differences in the effects of predictors between the 2 groups (Table 5). We found that the only significant difference is related to the effect of perceived barriers. The perceived barriers cannot influence nonusers’ continued use intentions, while they can significantly reduce the experience group’s intentions. Finally, when the model was tested in each subsample, we found that for experienced users, the effects of perceived threat severity and self-efficacy in using CT apps were nonsignificant. For the group with no prior experience, in addition to the nonsignificant effect of self-efficacy, the effect of perceived barriers was found to be nonsignificant.

**Table 5:** Results of post hoc analysis 2.

Predictors of intention	Path coefficients (exp^a^)	*P* value (no exp^b^)	Path coefficients (exp)	*P* value (no exp)	Path coefficients (difference)	*P* value (difference)
Perceived benefits of CT^c^ app use	0.42	<.001	0.42	<.001	<0.001	.99^d^
Perceived barriers of CT app use	−0.28	<.001	−0.05	.49^d^	−0.23	.01
COVID-19 threat severity	0.07	.17^d^	0.15	.03	−0.09	.33^d^
COVID-19 threat susceptibility	0.09	.05	0.17	.03	−0.08	.34^d^
Self-efficacy to use CT apps	0.08	.09^d^	0.01	.90^d^	0.07	.43^d^
Cue to action (using CT apps)	0.26	<.001	0.43	<.001	−0.17	.08^d^

^a^Exp: user with prior CT app experience.

^b^No exp: users who have had no prior experience with CT apps.

^c^CT: contact tracing.

^d^Not significant.

## Discussion

### Principal Findings

This study aimed to improve the current knowledge of the predictors for the continued use of CT apps. To this end, we draw upon the HBM [38,39] to propose a research model that shows the effect of 6 predictors. Analysis of data collected from 362 individuals showed that perceived benefits, self-efficacy, perceived severity, perceived susceptibility, and cues to action positively predicted the continued use intentions of CT apps, while perceived barriers could reduce them. Furthermore, we tested the possible differences among individuals in the United States versus the United Kingdom and those who previously used CT apps versus those who did not. These analyses revealed that the influence of critical predictors is similar in both the US and UK groups, with 1 exception—the effect of COVID-19 threat susceptibility is significant for the UK group but not for the US group. In addition, we noticed that the only significant difference between users and nonusers is related to the effect of perceived barriers; perceived barriers may not influence nonusers’ continued use intentions, while they can significantly reduce experienced users’ intentions.

Prior research has provided mixed results regarding the relationship between whether people are worried about COVID-19 and their intention to use a CT app [15]. Some studies reported that perceived health threat is positively associated with acceptance of CT app [37]. In addition, people who perceived lower health threats from COVID-19 tend to have a lower intention to embrace CT technology [16,37]. However, a few other studies showed contrasting findings—that perceived severity and perceived susceptibility were not related to the motivation for using CT apps [26,27]. In our study, when considering the entire sample, we found that perceived severity and susceptibility of COVID-19 could positively predict continued use intention. One possible explanation is that the prior studies found nonsignificance of a health threat in terms of CT app adoption intention at the early stage of the crisis when the government issued stay-at-home orders and mandated mask wearing. These measures limit people’s contact with others, which could lead them to think that they are less susceptible to the virus. In contrast, at the time of our study, confinement measures and mandatory mask wearing were lifted, while a new highly transmissible variant (omicron) quickly spread in the community; such a situation could have influenced existing users’ threat appraisal and intentions to continue using their CT app. Another explanation could be that individuals perceived adoption and continued use quite differently and considered different factors important in their decisions [63].

Regarding the impact of perceived benefits, prior studies found that social benefits (eg, using the app for the benefit of society) motivated CT app adoption [10,20,64,65]. However, mixed results were reported about the effect of personal benefits [19,66]; for example, as pointed out by Trang et al [66], compared with perceived social benefits, personal benefits seem to minimize the willingness to use a CT app among both critical and undecided respondents. In our study, we found that both perceived social benefits and personal benefits contributed to higher continued use intention of CT apps.

Many studies have highlighted the significant relationship between perceived barriers and CT app adoption intention [19,20,67]. A prominent perceived barrier for users is their concern about privacy issues raised by the app [26,34]. For example, the fear of data breaches or data misuse [18,27] and the fear of surveillance by the government [10,68] are significant barriers that prevent citizens from adopting CT apps. Consistent with prior findings, we found that perceived barriers can reduce individuals’ willingness to continue using CT apps. This finding of the negative impact of perceived privacy on the uptake and continued use of CT apps highlighted the importance of informing users of how their privacy and data security are protected within the app. Privacy concern was also one of the motives behind developing and launching decentralized CT apps, which store and analyze personal data on users’ devices, while the central server plays only a minor role in the CT process [69]. In addition, some CT methods have been proposed to use data-minimizing solutions such that they do not use user location data [70]. Future studies can investigate whether decentralized CT systems and clarifying data access can address these privacy concerns.

Individuals’ beliefs in their competence to use the app (self-efficacy) have been associated with their acceptance or intention to use a CT app in multiple studies [19,26,27]. In particular, self-efficacy for app use decreases with age, as the younger population has more experience with digital technologies such as smartphone apps [26]. Similar to the results reported in prior work, our study found that having self-efficacy to use CT apps can positively explain continued use intentions. Future work could examine how to promote app uptake among the population with low technical proficiency and experience. This effort may also contribute to bridging the digital divide.

Our study found a positive relationship between cues to action (eg, exposure to information) and CT app continued use. This finding indicates that more (traditional and on the web) media coverage of CT apps can enhance their continued use. This finding aligns with prior work reporting that people’s media consumption could influence their attitudes and intentions toward the app [71]. In this regard, more research can be conducted to investigate and analyze media coverage and web-based discussions on social media to gain insights into concerns and questions about the app. These insights can be used to inform app developers and governments to better communicate the usefulness and effectiveness of CT apps.

Our study also revealed between-country differences, even though the United States and the United Kingdom have followed similar COVID-19 measures. For example, perceived susceptibility has a significant effect on the UK group but not on the US group. In addition, we found nonsignificant effects for COVID-19 threat severity and susceptibility in the US group. This finding could be related to the upsurge of COVID-19 cases in the United Kingdom at the time of data collection, and United Kingdom residents may have felt more susceptible to the threat of COVID-19 during that period.

Finally, we observed differences in the effects of predictors between users and nonusers. More specifically, the perceived barriers were not associated with nonusers’ continued use intentions, but they could significantly reduce the intentions of the experienced group. One explanation for this difference could be that those who are yet to use CT technology cannot have an assessment of the potential challenges they may face, such as privacy and security, and this construct was not perceived as important in nonusers’ decisions. As privacy concerns may not be the primary reason for nonadoption of CT apps among nonusers, future work needs to investigate the primary facilitators and barriers for nonusers to adopt and start using CT apps.

Overall, our results revealed that the factors contributing to the adoption of CT apps also play an essential role in existing users’ intention to continue app use. More specifically, perceived benefits, self-efficacy, perceived severity, perceived susceptibility, and cues to action can motivate the continued use of CT apps, whereas perceived barriers could reduce an individual’s intention to continue using a CT app. Another interesting finding is that perceived barriers could significantly reduce experienced user’s continued use intentions, but this predictor had a limited influence on nonusers’ intentions.

### Practical Implications

Our study has several practical implications. First, as perceived social and personal benefits contributed to higher continued use intention of CT apps, these apps should be designed to continuously inform users about the potential social and personal benefits to ensure the effective use of CT apps in the long run. For example, these apps could present timely and updated informational resources (eg, advice on self-isolation, preventive, and testing options) to inform and assist users according to the COVID-19 trend (eg, the emergence of a new variant). In addition, a clear description of benefits to the user and society [66] as well as basic statistics that help users understand how the app aids people and society combat COVID-19 [34] are worth exploring. Second, more media coverage of CT apps might lead to continued use; as such, marketing and promoting such tools on both traditional media outlets and popular social media platforms (eg, Instagram or TikTok) could be helpful. This is similar to recent suggestions [72] about viable CT promotional strategies, such as using public health experts, independent privacy experts, and celebrities to endorse using these apps. In addition, over the past few years, we have witnessed much misinformation about COVID-19; the government and social media company decision makers should target misinformation about CT apps and provide proven factual and scientific information about these apps. Third, given that the perception of barriers (such as loss of privacy) could significantly reduce continued use, it might be useful to provide personalized options according to each user’s preference for data sharing. For example, CT apps could offer an opt-in feature for users who are willing to contribute more location and personal data to obtain more useful features, while allowing those who are more concerned about data privacy risks to provide minimum data access yet be able to use the basic CT service [34].

### Limitations and Future Work

There are several limitations. First, we had little representation of older adults and those with limited education levels. As age and education level could be associated with the use of CT apps, data collection in the future would benefit from a booster sample of underrepresented and minority participants with a low level of education and technology proficiency to better understand inequalities across diverse populations. Second, we conducted a cross-sectional study rather than a longitudinal study. Future work should examine the facilitators of and barriers to the long-term use of CT apps. Finally, other models and constructs may provide important insights into the continued use of CT apps. Future studies can contribute to the understanding of CT apps’ continued use and adoption by adopting other models.

### Conclusions

The effectiveness of CT apps depends on not only their uptake by citizens but also their continued use during the COVID-19 pandemic. Our work contributes to the knowledge of facilitators and barriers in determining an individual’s continued use intention of CT apps. We found that perceived benefits, self-efficacy, perceived severity, perceived susceptibility, and cues to action have significant positive impacts on the continued use intentions of CT apps, while perceived barriers can reduce such intentions. Further analyses revealed some degree of difference between users and nonusers. Those insights can be used by governments, technology companies, and media outlets to better promote the adoption and continued use of CT apps.

## Abbreviations

CT: contact tracing
HBM: health belief model
MGA: multigroup analysis
